# Clinical impact of TP53 mutation status on survival outcomes in metastatic colorectal cancer

**DOI:** 10.1590/1806-9282.20250998

**Published:** 2026-03-30

**Authors:** Oğuzhan Yıldız, Hakan Şat Bozcuk, Melek Karakurt Eryılmaz, Murat Araz, Ali Fuat Gürbüz, Mahmut Selman Yıldırım, Mehmet Artaç

**Affiliations:** 1 Necmettin Erbakan University, School of Medicine, Department of Medical Oncology – Konya, Türkiye.; 2 Medical Park Antalya Hospital, Department of Medical Oncology – Antalya, Türkiye.; 3 Necmettin Erbakan University, School of Medicine, Department of Medical Genetics – Konya, Türkiye.

**Keywords:** Colorectal neoplasms, TP53 protein, Survival analysis, Progression-free survival, Prognosis

## Abstract

**BACKGROUND:**

The prognostic value of TP53 mutations in metastatic colorectal cancer remains unclear owing to inconsistent findings in the literature. Given its central role in tumor biology, clarifying the impact of TP53 status on survival outcomes is clinically relevant.

**METHODS:**

This retrospective cohort study included 115 patients with metastatic colorectal cancer who underwent TP53 mutational analysis using next-generation sequencing (KAPA HyperPETE Pan Cancer Panel). Eligible patients were aged ≥18 years, had histologically confirmed metastatic colorectal cancer, and received first-line systemic therapy. Patients with incomplete clinical or molecular data were excluded. Based on mutation profiles, those with RAS or BRAF mutations received anti-VEGF therapy, whereas patients with wild-type tumors received anti-EGFR treatment. Patients were stratified according to TP53 mutation status to evaluate differences in clinical outcomes.

**RESULTS:**

Among the 115 patients included, 78 (67.8%) harbored TP53 mutations and 37 (32.2%) had wild-type TP53. The median progression-free survival was significantly longer in the TP53-mutant group (18.6 vs. 10.0 months; p=0.002). The median overall survival was also numerically longer in the TP53-mutant group (32.9 vs. 30.3 months), although this difference was not statistically significant (p=0.114). In the univariate analysis, TP53 mutation was associated with improved progression-free survival (HR 0.479; 95%CI 0.294–0.779; p=0.003). This association remained independently significant in the multivariate analysis (HR 0.477; 95%CI 0.289–0.787; p=0.004). None of the other variables consistently predicted survival.

**CONCLUSION:**

TP53 mutations appear to be an independent prognostic marker for prolonged progression-free survival in patients with metastatic colorectal cancer. Although the difference in overall survival was not statistically significant, these findings warrant further validation in prospective studies to confirm the prognostic utility of TP53 in therapeutic stratification.

## INTRODUCTION

Colorectal cancer (CRC) is the third most common cancer and the third leading cause of cancer-related death worldwide. Each year, approximately 1.3 million new cases are diagnosed, with 20% presenting with metastatic disease. The 5-year survival rate in this population is approximately 14%^1^. Systemic therapy—comprising cytotoxic chemotherapy, immunotherapy, biologic agents targeting growth factor pathways, or their combinations—forms the backbone of metastatic CRC treatment. Recent trials have emphasized the importance of tailoring therapies based on pathological and molecular profiles^2^. The TP53 gene, first identified as mutated in human cancers in 1989^3^, remains one of the most frequently altered genes in CRC and is found in approximately 34% of proximal and 45% of distal or rectal tumors^4^. TP53 is a key regulator of DNA repair, cell cycle control, senescence, metabolism, and apoptosis. However, its high mutation frequency and the diversity of alterations make it difficult to target therapeutically, particularly as standard treatments often rely on intact TP53 function to trigger cell death^5,6^.

Despite its biological significance, data on the prognostic and predictive value of TP53 mutations in CRC remain limited and inconsistent. Therefore, this study aimed to investigate the association between TP53 mutation status and survival outcomes in patients with metastatic CRC.

## METHODS

### General considerations

This retrospective study included patients diagnosed with metastatic CRC (mCRC) who received treatment at the Department of Medical Oncology, Necmettin Erbakan University Faculty of Medicine Hospital, between July 7, 2017, and July 1, 2023. Eligible patients were ≥18 years of age, had a confirmed diagnosis of mCRC, and underwent TP53 mutational analysis. Ethical approval was obtained from the Institutional Ethics Committee.

TP53 mutation status was determined using next-generation sequencing (NGS) with the KAPA HyperPETE Pan Cancer Panel (96 reactions), which employs primer extension target enrichment (PETE) technology. This method ensures high sensitivity and specificity, enabling accurate detection of somatic mutations in low-quality DNA, including formalin-fixed paraffin-embedded (FFPE) samples. The panel’s comprehensive design allowed for reliable profiling of TP53 mutations within a broad oncogenic framework.

First-line treatment selection was guided by molecular status: patients with Kirsten rat sarcoma viral oncogene homolog (KRAS), NRAS, or BRAF mutations typically received anti-VEGF therapy, while anti-EGFR therapy was preferred in RAS/BRAF wild-type cases. PFS and OS were defined as the time from the initiation of first-line therapy to disease progression and death, respectively. Kaplan-Meier estimates were used for survival analysis, and comparisons were made using the log-rank test. Cox proportional hazards models were applied for univariate and multivariate analyses of prognostic factors. Variables with p<0.05 in univariate analysis were entered into the multivariate model. Hazard ratios (HRs) and 95%CIs were calculated. Statistical significance was defined as a two-sided p<0.05. All statistical analyses were performed using SPSS version 21.0.

## RESULTS

**Figure 1:**
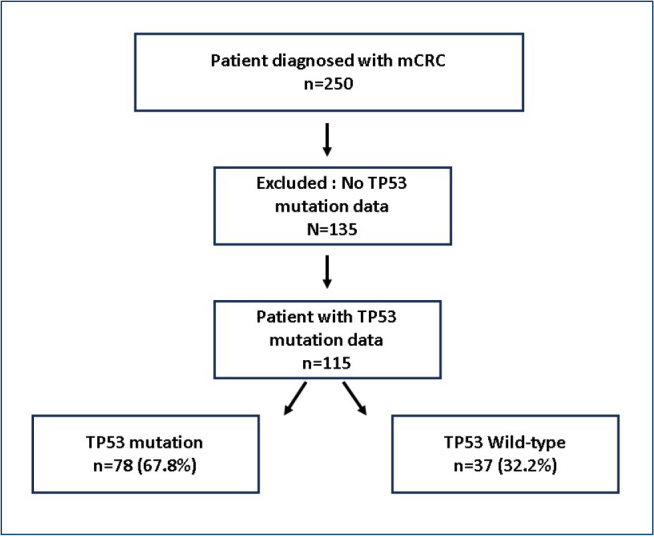
Flow diagram of patient selection and inclusion.

A total of 250 patients diagnosed with metastatic colorectal cancer were initially screened for eligibility. TP53 testing was not routinely performed during the study period; therefore, 135 patients without TP53 analysis were not eligible for inclusion. As a result, 115 patients with available TP53 mutational data were included in the final analysis. The flow diagram of patient selection is presented in Figure 1. The median age was 61.7 years, and 63.5% were ≤65 years. TP53 mutations were more frequent in younger patients than in older patients (74.0 vs. 57.1%; p=0.063). The mutation rate did not significantly differ by sex (68.4% in females vs. 67.5% in males; p=0.924), smoking status (68.9% in smokers vs. 67.1% in nonsmokers; p=0.845), metastatic status at diagnosis (67.2 vs. 68.8%; p=0.858), KRAS mutation status (63.8 vs. 71.9%; p=0.350), mismatch repair (MMR) status (85.7% in deficient mismatch repair [dMMR] vs. 65.3% in proficient mismatch repair [pMMR]; p=0.126), or presence of liver metastasis (69.6 vs. 63.9%; p=0.542). TP53 mutations were significantly more frequent in left-sided tumors than in right-sided tumors (73.9 vs. 48.1%; p=0.012).

Among patients harboring TP53 mutations (n=78), overall survival (OS) was evaluated according to KRAS mutation status. The median OS was 32.0 months (95%CI 29.1–35.0) in the TP53/KRAS co-mutated group (n=37) and 37.8 months (95%CI 22.4–53.1) in the TP53-mutant/KRAS wild-type group (n=41). Although numerically longer survival was observed in the KRAS wild-type subgroup, the difference was not statistically significant (log-rank p=0.692). Within the NRAS and BRAF subgroups of the TP53-mutant cohort (n=78), only five patients harbored concomitant NRAS mutations and four had BRAF mutations. Owing to the very small sample sizes and limited number of events in these subgroups, comparative survival analyses were not performed to avoid unstable estimates; therefore, only descriptive data are presented. Among TP53-mutant patients (n=78), OS was also evaluated according to primary tumor location.

Among patients harboring TP53 mutations (n=78), tumor-sidedness analysis revealed a marked predominance of left-sided primaries. Specifically, 65 patients (83.3%) had left-sided tumors, whereas only 13 patients (16.7%) had rightsided tumors. TP53 mutations were thus significantly more frequent in left-sided than in right-sided colorectal cancers (73.9 vs. 48.1%; p=0.012). In survival analysis, the median OS was 32.9 months (95%CI 24.2–41.5) for patients with left-sided TP53-mutant tumors and 27.0 months (95%CI 20.8–40.4) for those with right-sided tumors. Although a numerically longer OS was observed in the left-sided group, the difference was not statistically significant (log-rank p=0.783). These findings indicate that, despite the unequal distribution favoring leftsided disease, tumor sidedness did not exert a significant prognostic impact on survival within the TP53-mutant subgroup. In the subset of TP53-mutant patients with left-sided tumors (n=65), OS was analyzed according to KRAS mutation status. The median OS was 31.9 months (95%CI 20.0–43.7) in KRAS-mutant cases (n=31) and 40.7 months (95%CI 21.6– 59.9) in KRAS wild-type cases (n=34). Although numerically higher survival was observed in the KRAS wild-type group, the difference was not statistically significant (log-rank p=0.358).

Following the exclusion of patients with BRAF mutations or dMMR tumors (n=12), survival analysis was repeated in the remaining 66 TP53-mutant cases (right-sided, n=11; left-sided, n=55). The median OS demonstrated no significant difference between right- and left-sided tumors (log-rank p=0.974), suggesting that tumor location does not have a prognostic impact, even after excluding these biologically distinct subgroups.

In the univariate Cox regression analysis, TP53 mutation was significantly associated with improved progression-free survival (PFS) (HR 0.479; 95%CI 0.289–0.787; p=0.003). Liver metastasis showed a trend toward shorter PFS (HR 0.790; 95%CI 0.578–1.079; p=0.098), whereas MMR deficiency demonstrated a borderline association (HR 0.762; 95%CI 0.525–1.106; p=0.174). Other factors, including smoking status, KRAS mutation, sex, tumor location, and presence of metastasis at diagnosis, were not significantly associated with PFS. In the multivariate analysis, TP53 mutation remained an independent predictor of prolonged PFS (HR 0.700; 95%CI 0.543–0.901; p=0.004). Liver metastasis (HR 0.627; 95%CI 0.361–1.087; p=0.105), MMR status (HR 0.784; 95%CI 0.348–1.763; p=0.555), and smoking status (HR 1.545; 95%CI 0.937–2.546; p=0.088) were not independently associated with PFS (Table 1).

**Table 1:** Univariate and multivariate Cox regression analyses of clinicopathological variables associated with progression-free and overall survival.

Correlates of progression of free survival				
Variable	Univariate analysis HR (95%CI)	p-value	Multivariate analysis HR (95%CI)	p-value
p53: mutant vs. wild	0.479	0.003	0.700	0.004
Gender: female vs. male	1.013	0.921		
Smoking: yes vs. no	1.193	0.158	1.545	0.088
Metastasis at diagnosis: yes vs. no	0.999	0.993		
Tumor location: right vs. left	0.945	0.715		
MMR status: dMMR vs. pMMR	0.762	0.174	0.880	0.555
KRAS status: mutant vs. wild	1.038	0.753		
Liver metastasis: yes vs. no	0.790	0.098	0.800	0.105
**Correlates of overall survival**				
p53: mutant vs. wild	0.681	0.116	0.691	0.131
Gender: female vs. male	1.111	0.412		
Smoking: yes vs. no	1.226	0.108	1.193	0.063
Metastasis at diagnosis: yes vs. no	0.82	0.109	0.946	0.094
Tumor location: right vs. left	0.769	0.376		
MMR status: dMMR vs. pMMR	0.852	0.395		
KRAS status: mutant vs. Wild	1.11	0.381		
Liver metastasis: yes vs. No	1.163	0.258		

HR: hazard ratio; CI: confidence interval; MMR: mismatch repair; dMMR: deficient mismatch repair; pMMR: proficient mismatch repair; KRAS; Kirsten rat sarcoma viral oncogene homolog.

In the univariate analysis, trends toward improved OS were observed in patients without liver metastases (HR 1.163; 95%CI 0.900–1.502; p=0.258), with wild-type TP53 (HR 0.681; 95%CI 0.420–1.104; p=0.116), among nonsmokers (HR 1.226; 95%CI 0.943–1.595; p=0.108), and in those without metastatic disease at diagnosis (HR 0.820; 95%CI 0.634–1.060; p=0.109), although the differences were not statistically significant. Sex, tumor location, MMR status, and KRAS mutation were not associated with OS. The multivariate analysis included TP53 status, smoking, and metastatic presentation (p<0.20 in the univariate analysis). While none were independently significant, smoking (HR 1.618; 95%CI 0.973–2.690; p=0.063) and the absence of metastasis at diagnosis (HR 0.656; 95%CI 0.401–1.074; p=0.094) showed trends toward significance. TP53 mutations were also associated with a non-significant trend toward better OS (HR 0.691; 95%CI 0.427–1.117; p=0.131). The overall model was statistically significant (p=0.046), indicating a collective prognostic contribution (Table 1).

**Figure 2:**
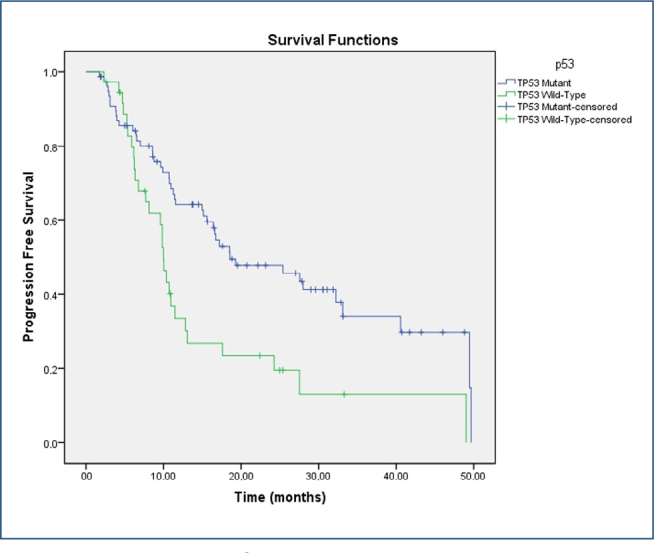
Progression-free survival by TP53 mutation status comparison of progression-free survival between TP53-mutant and wild-type groups. Median values (with 95%CI) are reported. Patients harboring TP53 mutations demonstrated significantly longer progression-free survival (log-rank p=0.002).

The median PFS was 15.6 months (95%CI 10.4–20.8) in the overall cohort. Patients with TP53-mutant tumors had a median PFS of 18.6 months (95%CI 8.2–28.9), whereas those with wild-type TP53 had a median PFS of 10.0 months (95%CI 9.2–10.8) (log-rank p=0.002) (Figure 2).

**Figure 3:**
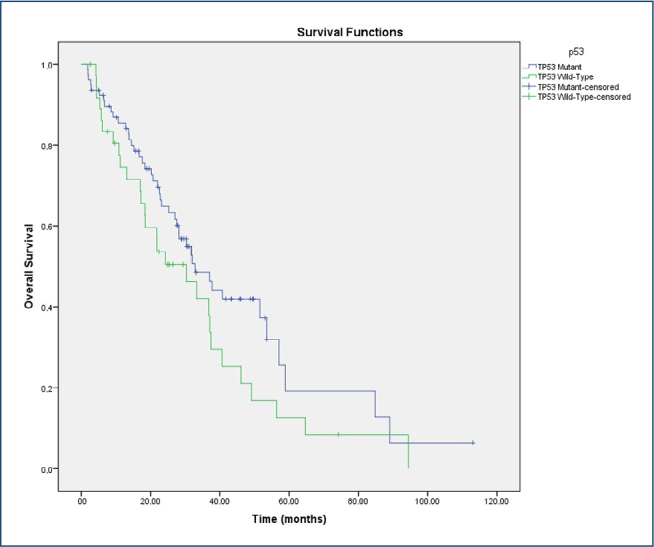
Overall survival by TP53 mutation status comparison of overall survival between TP53-mutant and wild-type groups. Median values (with 95% confidence intervals) are reported. Although patients with TP53 mutations exhibited numerically longer overall survival, the difference did not reach statistical significance (log-rank p=0.114).

The prognostic impact of TP53 mutations on OS and PFS was evaluated using Kaplan-Meier curves and Cox proportional hazards regression analysis. According to the Kaplan-Meier analysis, patients harboring TP53 mutations exhibited significantly longer progression-free survival compared with those with wild-type TP53 (median PFS: 18.6 months, 95%CI 8.2–28.9 vs. 10.0 months, 95%CI 9.2–10.8; logrank p=0.002). This finding was further supported by the Cox regression analysis, in which TP53 mutation was identified as an independent prognostic factor for improved PFS (HR 0.700, 95%CI 0.543–0.901; p=0.006). The median OS was 32.0 months (95%CI 24.0–40.1) in the overall cohort. Patients with TP53-mutant tumors had a median OS of 32.9 months (95%CI 23.3–42.4), whereas those with wild-type TP53 had a median OS of 30.3 months (95%CI 16.6–44.0). This difference was not statistically significant (log-rank p=0.114) (Figure 3).

While Kaplan-Meier analysis demonstrated a numerically longer OS in the TP53-mutant group, this difference was not statistically significant (log-rank p=0.114). In contrast, the univariate Cox regression model yielded a HR of 1.468 (95%CI not shown; p=0.116) for the TP53-mutant versus wild-type group, suggesting a non-significant trend toward worse survival in mutant cases. The discrepancy between visual and model-based interpretations may be attributed to methodological differences between risk modeling and time-to-event estimation.

Consistently, the difference in overall survival between the groups did not reach statistical significance, and Cox regression analysis likewise did not demonstrate a significant association between TP53 mutations and OS (HR 0.681; 95%CI 0.411–1.127; p=0.116). These findings suggest that TP53 mutation has significant prognostic value for PFS but not for OS. The absence of a statistically significant association with OS, despite a numerical trend, may reflect the influence of additional clinical factors or subsequent lines of therapy that mitigate the long-term impact of TP53 status.

## DISCUSSION

In this retrospective cohort study, we demonstrated that TP53 mutation status is significantly associated with PFS in patients with mCRC. Specifically, patients harboring TP53 mutations had significantly longer median PFS than those with wild-type TP53 (18.6 vs. 10.0 months; p=0.002), and TP53 mutation remained an independent prognostic factor in multivariate analysis. In contrast, OS was numerically longer in the TP53-mutant group, but the difference was not statistically significant (32.9 vs. 30.3 months; p=0.114). These findings suggest a potential role for TP53 mutations as prognostic biomarkers for disease control rather than for long-term survival in patients with mCRC. Our study adds to this heterogeneous body of evidence by demonstrating that TP53 mutations, despite their known association with poor prognosis, may be linked to delayed disease progression under first-line systemic treatment. The higher-than-expected frequency of TP53 mutations observed in our study (67.8%) may be explained by several factors. First, our cohort exclusively comprised patients with metastatic disease, who are more likely to harbor aggressive molecular features, including TP53 alterations. Second, the use of NGS-based testing platforms in our institution might have enhanced mutation detection sensitivity compared to older sequencing methods.

The prolonged PFS observed in patients with TP53 mutations may be associated with tumor location and treatment distribution. In the present study, the distribution of TP53 wild-type and mutant cases was found to be balanced in right colon tumors, while the majority of left colon tumors were observed in TP53-mutant patients. The higher frequency of TP53 mutations in the left colon, the greater use of anti-EG-FR-based therapy in the first line in these patients, and their generally better response to these therapies may provide a rationale for the observed survival advantage. Furthermore, the functional heterogeneity of TP53 alterations may also play a role, as gain-of-function mutations can enhance chemosensitivity by increasing genomic instability, whereas loss-of-function mutations may promote resistance mechanisms. The absence of a significant OS difference despite prolonged PFS suggests that the initial therapeutic advantage could have been attenuated by treatment heterogeneity, subsequent lines of therapy, and potential selection bias.

TP53 mutations are associated with unfavorable prognostic features in many cancer types. The presence of TP53 mutations correlates with increased tumor aggressiveness, resistance to apoptosis, and worse clinical outcomes^7^. In a meta-analysis, Barry Iacopetta reported that TP53 mutations were associated with poorer survival outcomes in colorectal cancer patients^8^. Recent evidence suggests that TP53 mutations may be more complex in advanced and metastatic disease. A considerable number of studies have indicated that tumors with TP53 mutations may exhibit a superior response to systemic chemotherapy or targeted therapies under specific conditions. This phenomenon may be attributable to alterations in DNA damage response mechanisms^9,10^. These discrepancies may be attributed to the heterogeneity in the study populations, differences in treatment regimens, or variability in the molecular subtypes of TP53 mutations. One possible explanation for the observed improvement in PFS may be related to the interaction between the TP53 mutational status and specific chemotherapeutic or targeted agents, which may sensitize tumors to treatment-induced stress and apoptosis. For instance, the biological impact of TP53 alterations may differ depending on whether the mutation confers a gain-of-function or loss-of-function effect, potentially affecting the tumor microenvironment in distinct ways. The lack of statistically significant improvement in OS, despite prolonged PFS, suggests that subsequent lines of treatment and tumor evolution may diminish the early therapeutic benefit conferred by TP53 mutations^11^. A review of the extant literature reveals a paucity of consensus regarding this issue. For example, Wang et al. reported significantly shorter disease-free survival (DFS) and OS in patients with TP53-positive mCRC, whereas Said et al. demonstrated prolonged mPFS in patients with TP53 mutations treated with bevacizumab^12,13^. In contrast, Munro et al. and Kim et al. found no significant association between TP53 abnormalities and chemotherapy outcomes. Interestingly, Kim et al. reported that TP53 mutations were linked to shorter OS, but not to relapse-free survival^14^. Our findings are more consistent with those of Said et al.^13^, suggesting that TP53 mutations may predict better disease control in the metastatic setting, particularly under anti-angiogenic therapy. In contrast, the adverse prognostic effect reported by Wang et al.^12^ likely reflects differences in disease stage and therapeutic context, as their cohort predominantly included localized CRC patients receiving adjuvant chemotherapy. Moreover, the biological heterogeneity of TP53 alterations, which may include loss-of-function or gain-of-function mutations, together with the presence of co-occurring alterations such as KRAS, BRAF, or MMR deficiency, may contribute to the variability in treatment responses and survival outcomes reported across studies. These distinctions emphasize that the prognostic implications of TP53 mutations should be interpreted in the context of disease stage, treatment modality, and molecular profile.

A notable finding of our study was the discrepancy between improved PFS and the absence of a corresponding OS benefit in TP53-mutant patients. This observation likely reflects the complex interplay between early treatment sensitivity and subsequent resistance mechanisms. TP53 alterations may initially enhance cytotoxic efficacy by impairing DNA damage repair and promoting apoptosis under treatment-induced stress, leading to prolonged disease control in the early phase of therapy. However, the same genomic instability that confers early chemosensitivity can facilitate clonal evolution, intratumoral heterogeneity, and the emergence of resistant subclones over time, ultimately offsetting any long-term survival advantage. In addition, variability in post-progression therapies and the limited sample size may have further attenuated the OS difference between TP53-mutant and wild-type groups. These findings suggest that TP53 mutations may confer a transient therapeutic advantage rather than a durable survival benefit, highlighting the need for longitudinal studies exploring the molecular dynamics of TP53-driven tumor evolution during treatment.

In our TP53-mutant cohort, there was no significant difference in OS between the KRAS wild-type and KRAS-mutant groups. Although previous studies have reported that KRAS/TP53 co-mutations are associated with poor prognosis, our findings suggest that this relationship may be influenced by other clinicopathological factors such as tumor location^15,16^. Tumor sidedness did not significantly impact survival outcomes among patients with TP53 mutations. The majority of patients in this subgroup had left-sided tumors, which are generally associated with a more favorable prognosis. It is possible that the presence of TP53 mutations, known to influence tumor biology and treatment response, may modulate or even neutralize the typical prognostic effects of tumor location^17^. In our study, a limited number of patients harbored both TP53 and BRAF mutations. Although each is generally considered a poor prognostic factor, some findings in the literature suggest that survival may be better than expected when these mutations co-occur. This raises the possibility that the interaction between these mutations may affect tumor biology in a distinct manner. However, due to the limited number of cases, no statistical analysis could be performed. Therefore, large-scale, prospectively designed studies with detailed molecular characterization are needed to better understand the prognostic factors influencing survival in patients harboring high-risk mutations such as TP53 and BRAF^18^.

The biological mechanisms that may influence these findings require further evaluation. TP53 has been demonstrated to play a significant role in the regulation of various cellular processes, including DNA repair, cell cycle regulation, and apoptosis. Mutations in TP53 can result in elevated genomic instability^18,19,20^. Some TP53 mutations can impair the ability of tumor cells to respond to DNA damage, thereby increasing their susceptibility to chemotherapy-induced toxicity. These conditions may explain the prolonged PFS observed in TP53 mutation patients.

Our study has several limitations. First, the retrospective design may have introduced selection and information bias. Second, the relatively small sample size may limit the generalizability of our findings, particularly in subgroup analyses. The present study is not without its limitations. First, the retrospective design may have introduced selection and information bias. It is important to note that TP53 analysis was not included in the routine reflex testing panel (which included KRAS, NRAS, and BRAF) during the study period at our institution. Consequently, patients without available TP53 results were not excluded on the basis of missing data, but rather were not tested and thus not eligible for inclusion in the cohort. Second, the relatively limited sample size, particularly within molecular subgroups such as BRAF- or NRAS-mutant TP53 cases, constrained the statistical power of subgroup analyses. Consequently, the findings observed in these subsets should be regarded as descriptive rather than inferential and interpreted with caution.

Third, although TP53 mutations were predominantly observed in left-sided tumors, survival outcomes among TP53-mutant patients did not differ significantly according to tumor location. This finding suggests that the prognostic effect of TP53 mutation on PFS is not attributable to the typical survival advantage seen in left-sided colorectal cancers. In our cohort, left-sided tumors were more often treated with anti-EGFR– based regimens, whereas right-sided tumors predominantly received anti-VEGF therapy. However, comparable outcomes between these subgroups indicate that the prognostic value of TP53 mutation is likely independent of both tumor sidedness and the class of targeted agent administered. Given the limited number of right-sided cases, these results should still be interpreted with caution and validated in larger, prospectively designed studies. A sensitivity analysis excluding patients with BRAF mutations or dMMR tumors was also conducted to minimize potential molecular confounding. No meaningful difference in survival outcomes was observed according to tumor sidedness after these biologically distinct subgroups were excluded. These findings indicate that the comparable outcomes between right- and left-sided TP53-mutant colorectal cancers are not attributable to the unequal distribution of BRAF or MMR status. This supports the interpretation that TP53 mutation exerts an independent prognostic effect, irrespective of tumor location or coexisting molecular alterations.

Finally, the absence of standardized data on post-progression treatments limits our ability to fully assess their impact on OS outcomes. Clinically, our results suggest that TP53 mutation status could be incorporated into risk stratification models to predict disease control in patients with mCRC initiating systemic therapy. Identifying patients who are likely to achieve prolonged PFS may assist in optimizing treatment planning and surveillance strategies. However, further prospective studies with larger cohorts and comprehensive molecular profiling are necessary to validate these findings and elucidate the role of specific TP53 mutation subtypes in therapeutic responsiveness.

## CONCLUSION

Our study highlights the potential prognostic significance of TP53 mutations in predicting delayed disease progression among patients with metastatic colorectal cancer receiving systemic therapy. Although TP53 mutations were associated with improved PFS, their impact on OS remains uncertain. These findings underscore the need for continued investigation into the complex biological role of TP53 in colorectal cancer and support the integration of molecular biomarkers into personalized treatment strategies. Given the modest sample size and the exploratory nature of subgroup analyses, these results should be interpreted with caution. Future large-scale, prospectively designed studies are warranted to validate and expand upon these observations.

## Data Availability

The datasets generated and/or analyzed during the current study are available from the corresponding author upon reasonable request.
